# Gender-Specific Insights into Depression in Patients with Ischemic Heart Disease: Findings from a Pilot Study Using a Self-Developed Questionnaire

**DOI:** 10.3390/diseases12120320

**Published:** 2024-12-10

**Authors:** Laura Ioana Bondar, Brigitte Osser, Caius Calin Miuța, Denis Petran, Alexandru Ioan Baltean, Denis Bogdan Butari, Mariana Adelina Mariș, Ligia Elisaveta Piroș, Robert Almășan, Mihaela Gavrila-Ardelean, Liviu Gavrila-Ardelean, Mircea Ioachim Popescu

**Affiliations:** 1 Doctoral School of Biomedical Sciences, University of Oradea, 410087 Oradea, Romania; bondar.lauraioana@student.uoradea.ro (L.I.B.); brigitte.osser@uav.ro (B.O.); procardia_oradea@yahoo.com (M.I.P.); 2 Department of Biology and Life Sciences, “Vasile Goldiș” Western University of Arad, 310048 Arad, Romania; butari.denis-bogdan@uvvg.ro; 3 Faculty of Physical Education and Sport, “Aurel Vlaicu” University of Arad, 310130 Arad, Romania; denis.petran@uav.ro (D.P.); alexandru.baltean@uav.ro (A.I.B.); 4 Department of General Medicine, “Vasile Goldiș” Western University of Arad, 310048 Arad, Romania; maris.mariana@uvvg.ro (M.A.M.); piros.ligia@uvvg.ro (L.E.P.); almasan.robert@uvvg.ro (R.A.); 5 Faculty of Educational Sciences Psychology and Social Work, “Aurel Vlaicu” University of Arad, 310130 Arad, Romania; mihaela.gavrila@uav.ro; 6 Prosthetic Dentistry, Faculty of Dental Medicine, Western University ’Vasile Goldis’, 310130 Arad, Romania

**Keywords:** depression, healthcare interventions, ischemic heart disease, mental health

## Abstract

Background/Objectives: Ischemic heart disease (IHD) significantly affects mental health, with gender-specific differences being observed in psychological responses. This pilot study aimed to explore these differences in the demographic, clinical, psychological, psychiatric, and social profile of patients diagnosed with IHD. Methods: A descriptive, cross-sectional design was used, recruiting 183 adult patients diagnosed with coronary artery disease and depression at the Psychiatry Department of Arad County Emergency Hospital, Romania, between May 2021 and May 2024. Data were collected using a self-developed tool, named the Depression Assessment in Ischemic Heart Disease Questionnaire (DA-IHDQ), alongside standardized assessments. Statistical analysis was performed using JASP statistical software (Version 0.19.1, University of Amsterdam, Amsterdam, Netherlands), employing binomial and multinomial tests for categorical data, and Cronbach’s alpha was used to assess internal consistency. Results: This study found significant demographic differences, with female patients exhibiting higher levels of emotional distress and severe depression compared with the male subjects. Women reported greater social isolation and a stronger desire to seek for psychological or psychiatric support. Furthermore, a positive correlation between depression severity and physical symptoms was observed in both genders. Conclusions: These findings highlight the importance of recognizing gender-specific responses to IHD, emphasizing the need for tailored interventions in mental healthcare and cardiac rehabilitation. Future research should further explore these differences to enhance the understanding of the psychosocial/psychiatric aspects of IHD and improve patient outcomes.

## 1. Introduction

Ischemic heart disease (IHD) remains one of the leading causes of morbidity and mortality worldwide, accounting for one million deaths annually and placing a substantial burden on healthcare systems globally [1,2]. Characterized by reduced blood flow to the heart, often resulting from atherosclerosis, IHD can lead to serious complications, including myocardial infarction and heart failure [3]. Beyond the physical manifestations, the psychological and social repercussions of IHD are profound, with significant implications for patient quality of life and adherence to treatment protocols. Research indicates that patients with IHD frequently experience comorbid conditions, particularly depression, which can complicate disease management and worsen clinical outcomes [4,5,6].

Recent studies have shown that gender significantly influences the prevalence, progression, and prognosis of IHD [7]. Women are often diagnosed later than men, and their symptoms may not be as easily recognized by healthcare providers, leading to delayed diagnosis and less aggressive treatment [8]. Gender-specific differences in IHD are multifaceted, with research indicating that women often experience different symptomatology, have distinct responses to treatment, and exhibit unique emotional and psychological reactions to the diagnosis [9]. While men typically show a higher prevalence of physical symptoms such as chest pain, women are more likely to report non-cardiac symptoms such as fatigue and nausea [10]. Furthermore, women tend to experience higher levels of psychological distress, which may exacerbate their cardiovascular condition and hinder recovery [11]. This highlights the need for a gender-sensitive approach to IHD diagnosis and treatment [12,13].

Studies on depression in IHD patients have shown that depression is not only common but also contributes significantly to poorer clinical outcomes, including increased risk of heart failure, worse quality of life, and higher mortality rates [4]. Research indicates that depression affects women with IHD more severely, with women being more likely to experience major depressive episodes compared with men. This, in turn, affects their cardiac rehabilitation and adherence to treatment protocols [14]. The relationship between depression and IHD is complex, as depression may worsen the prognosis of IHD, while the challenges of managing IHD can also exacerbate depression. Therefore, effective management of depression in IHD patients, particularly female patients, is crucial [15,16].

The interplay between gender and IHD has become a focal point of investigation in recent years, as studies reveal distinct differences in the clinical presentation and psychological responses between male and female patients. Women with IHD are often underdiagnosed and undertreated, experiencing unique challenges related to their symptoms and psychiatric well-being [9,17]. Evidence suggests that women may exhibit higher levels of emotional distress, including symptoms of sadness, anxiety, and hopelessness, compared with their male counterparts [18,19]. Furthermore, female patients are more likely to report feelings of social isolation and a heightened desire for psychological or psychiatric support, highlighting the necessity for gender-sensitive treatment approaches [20].

Psychological distress in IHD patients, particularly depression and anxiety, is associated with greater social isolation, reduced social support, and lower quality of life. These mental health issues are often under-addressed, and there is growing recognition of the need to integrate psychological care into routine cardiovascular treatment. For women, these psychological burdens are compounded by social and cultural factors, including caregiving responsibilities and gendered expectations of health. Thus, the psychosocial impact of IHD is not only a medical concern but also a social one, requiring targeted interventions to improve both mental and physical health outcomes [19,21].

Emerging evidence suggests that anatomical factors, such as anterior chest wall conformation, may influence IHD prevalence and prognosis. For example, individuals with a concave-shaped thoracic structure, commonly observed in females with narrow antero-posterior thoracic diameters, have been shown to have a lower prevalence of IHD and more favorable prognoses. This anatomical feature is also linked to benign mitral valve prolapse and a higher prevalence of anxiety disorders, both of which could further impact cardiovascular health. These findings highlight the importance of considering personalized approaches to IHD diagnosis and treatment [22].

Despite these insights, there remains a notable gap in the literature concerning the specific demographic, clinical, psychological, psychiatric, and social profiles of IHD patients, particularly in relation to gender. Existing research often utilizes broad diagnostic tools that may not fully capture the unique experiences of male and female patients, potentially leading to misdiagnosis and inadequate treatment. The Depression Assessment in Ischemic Heart Disease Questionnaire (DA-IHDQ), a self-developed pilot tool, was designed to address this deficiency by providing a targeted and nuanced assessment of depression in patients diagnosed with IHD.

The rationale for this study stems from the recognition that traditional diagnostic approaches often fail to account for the gender-specific manifestations of IHD and associated mental health challenges. By employing the DA-IHDQ questionnaire, this study aims to address the gaps identified in previous studies by providing a comprehensive examination of the demographic, clinical, psychological, psychiatric, and social profiles of patients diagnosed with IHD. Furthermore, this study aims to elucidate the ways in which gender influences the experience of IHD, particularly concerning emotional distress, social isolation, and the need for psychological/psychiatric support.

This investigation is essential not only for enhancing the understanding of gender differences in IHD but also for informing clinical practices. Recognizing the gender-specific emotional, psychological, and social needs of IHD patients will enable clinicians to offer more tailored and effective interventions. By highlighting the specific needs of male and female patients, this study intends to facilitate the development of targeted interventions that address these differences. Integrating mental healthcare into cardiac rehabilitation programs may ultimately lead to improved treatment outcomes and quality of life for both male and female patients.

The purpose of this study is to investigate gender-specific differences in the profile of patients diagnosed with IHD, leveraging the DA-IHDQ questionnaire to determine the prevalence and severity of depressive symptoms among male and female patients. By investigating these disparities, the research seeks to improve clinical practices and optimize patient care. The findings are expected to contribute to a more comprehensive understanding of IHD, paving the way for targeted interventions that address the psychological and social challenges faced by patients. Ultimately, this study aspires to improve treatment outcomes and quality of life for both male and female patients with IHD, emphasizing the importance of integrating mental healthcare into cardiac rehabilitation programs. Through this investigation, the study aims to bridge the existing knowledge gap and encourage further research into the complex relationship between gender, psychological/psychiatric health, and cardiovascular disease.

## 2. Materials and Methods

### 2.1. Study Design and Setting

This pilot study used a descriptive cross-sectional design to investigate gender-specific differences in the demographic, clinical, psychological, psychiatric and social profile of patients diagnosed with IHD at the Psychiatry Department of Arad County Emergency Hospital, Romania. Data were collected from May 2021 to May 2024, targeting adult male and female patients diagnosed with IHD and referred for psychiatric evaluation.

### 2.2. Study Population

The study population included 183 patients, selected based on the inclusion and exclusion criteria corresponding to Table 1.

### 2.3. Depression Diagnosis

The diagnosis of depression was established according to *Diagnostic and Statistical Manual of Mental Disorders, Fifth Edition* (DSM-5) criteria through comprehensive anamnesis, heteroanamnesis, and a transactional analysis of each patient’s personal history, illness progression, and family history. To validate the findings from the newly developed DA-IHDQ questionnaire, standardized tools such as the Beck Depression Inventory and the Hamilton Depression Rating Scale were also administered, confirming the accuracy of DA-IHDQ by yielding similar scores.

### 2.4. Data Collection Instruments

Data were collected using DA-IHDQ, a self-developed questionnaire consisting of 20 questions divided into two sections:

- Demographic and clinical data: This section gathered personal and medical data, including gender, age, marital status, social status, environment of origin, type of diagnosed IHD, and associated risk factors such as inflammation markers, hypertension, hypercholesterolemia, hypertriglyceridemia, genetic factors, high-density lipoprotein (HDL), and low-density lipoprotein (LDL) cholesterol levels.
  ○ Hypertension: Diagnosed as an office systolic blood pressure (BP) of ≥140 mmHg or diastolic BP of ≥90 mmHg [23].
  ○ Hypercholesterolemia: Total cholesterol levels of >200 mg/dL. LDL cholesterol is the primary target for cardiovascular risk reduction, with recommended LDL targets based on cardiovascular risk, including <116 mg/dL for low-risk individuals, <100 mg/dL for moderate-risk individuals, <70 mg/dL for high-risk individuals, and <55 mg/dL for very high-risk individuals. For HDL cholesterol, normal levels are considered as >0 mg/dL for men and >50 mg/dL for women, as higher levels of HDL cholesterol are protective against cardiovascular diseases [24].
  ○ Hypertriglyceridemia: Defined as triglyceride levels greater than 150 mg/dL [24,25].
  ○ Genetic Factors: Family history of cardiovascular diseases, particularly IHD [26].
  ○ Inflammation markers: C-reactive protein (CRP) and erythrocyte sedimentation rate (ESR) levels were measured as indicators of inflammation [27,28].
- Psychological/psychiatric and physical condition: This section included 11 questions assessing the patient’s current mental and physical state, rated from 0 to 3 points, where 0 indicates the absence of symptoms and 3 indicates severe symptoms. The total possible score ranged from 0 to 33, with higher scores indicating greater severity of depressive symptoms. The scoring breakdown for depression grading is according to Table 2.

The final question specifically addressed the patient’s interest in receiving psychological or psychiatric assistance.

### 2.5. Reliability Testing

The internal consistency of the DA-IHDQ questionnaire was evaluated using **Cronbach’s alpha (α)**, a measure of how well the items in the questionnaire reliably assess the intended construct. Cronbach’s alpha values range from 0 to 1, with values above 0.7 generally being considered acceptable. In this study, the questionnaire demonstrated strong internal reliability with a Cronbach’s alpha of 0.915, indicating that the items were highly consistent in measuring depressive symptoms in IHD patients. However, as the DA-IHDQ is a self-developed tool, further external validation in larger populations is required.

### 2.6. Ethical Considerations

This study received ethical approval from the Arad County Clinical Hospital Ethics Committee (Approval No. 38/6 April 2021). Informed consent was obtained from all participants prior to data collection, ensuring confidentiality and adherence to the principles outlined in the Declaration of Helsinki.

### 2.7. Statistical Analysis

Data analysis was performed using JASP statistical software (Version 0.19.1, University of Amsterdam, Amsterdam, The Netherlands). Descriptive statistics summarized the demographic, clinical, psychological, psychiatric, and social data. Graphical distributions and frequency tables were used for visual data representation. The following statistical tests were utilized:

- Chi-square analysis: employed to evaluate associations between categorical variables, such as gender differences in demographic, clinical, and psychological characteristics, with statistical significance being set at *p* < 0.05.
- Binomial test: to evaluate whether the proportion of responses for certain variables deviated significantly from a hypothetical proportion (e.g., <0.5), particularly for imbalanced categorical data related to psychological and psychiatric symptoms.
- Multinomial: applied to evaluate probabilities across multiple categories, particularly when analyzing demographic and clinical variables across gender groups.
- Scale reliability statistics: Cronbach’s alpha was calculated to assess the internal consistency of the DA-IHDQ questionnaire, with a threshold of ≥0.7 being considered acceptable reliability.

### 2.8. Hypotheses of the Study

The aim of this pilot study is to explore the gender-specific differences in the demographic, clinical, psychological, psychiatric, and social profile of patients diagnosed with IHD. To guide this investigation, the following hypotheses were developed based on the existing literature and clinical observations:

1. Demographic differences: there are significant demographic differences between male and female patients diagnosed with IHD, including age, marital status, and social status.
2. Clinical presentation: male patients with ischemic heart disease are more likely to present with acute myocardial infarction (AMI), while female patients are more likely to suffer from stable or unstable angina.
3. Psychological impact: female patients with IHD report higher levels of emotional distress, including greater sadness and hopelessness, compared with male patients.
4. Depression severity: the severity of depression is significantly higher in female patients than in male patients.
5. Social isolation: female patients diagnosed with IHD experience higher levels of social isolation and withdrawal compared with male patients.
6. Work performance: both male and female patients report a decline in work performance following their diagnosis of IHD, but the decline is more pronounced in female patients.
7. Interest in psychological and psychiatric support: a higher proportion of female patients express a desire for psychological or psychiatric assistance compared with male patients following their diagnosis of IHD.
8. Correlation of depression and physical symptoms: there are a significant correlation between the severity of depressive symptoms and the reporting of physical symptoms in both male and female patients with IHD.
9. Inflammatory effects: elevated inflammation levels in patients with IHD are associated with higher rates of depression and anxiety, negatively impacting their mental health and overall well-being.

## 3. Results

### 3.1. Assessment of Patient Personal and Medical Data

Table 3 presents the results of multinomial tests performed to examine the association between different variables (age, social status, marital status, type of IHD, and IHD onset) and gender. The breakdown of the main statistics is as follows:

- Age
  ○ Female (χ^2^ = 33.429, df = 2, *p* < 0.001): there is a statistically significant association between age and female gender; the *p*-value of <0.001 indicates that the age distribution is significantly different in females.
  ○ Male (χ^2^ = 7.364, df = 1, *p* < 0.001): similarly, age is significantly associated with male gender, suggesting a different age distribution in this group as well.
- Social Status
  ○ Female (χ^2^ = 32.310, df = 4, *p* < 0.001): there is a significant association between social status and gender among female patients, implying that social status varies significantly in this group.
  ○ Male (χ^2^ = 79.939, df = 4, *p* < 0.001): the social status distribution is also significantly different among male patients, with a highly significant *p*-value.
- Marital Status
  ○ Female (χ^2^ = 15.643, df = 2, *p* < 0.001): marital status is significantly associated with gender, indicating differences in marital status distribution among females.
  ○ Male (χ^2^ = 110.899, df = 3, *p* < 0.001): marital status shows a strong association with gender in males, reflected by the highly significant *p*-value.
- Type of IHD
  ○ Female (χ^2^ = 26.762, df = 3, *p* < 0.001): there is a significant association between the type of IHD and female gender.
  ○ Male (χ^2^ = 45.283, df = 3, *p* < 0.001): for males, the type of IHD is also significantly associated with gender, showing significant differences in the types of IHD diagnosed among male patients.
- IHD Onset:
  ○ Female (χ^2^ = 62.152, df = 5, *p* < 0.001): In females, a significant association is observed between IHD onset and gender, indicating different patterns of IHD onset in women.
  ○ Male (χ^2^ = 2.548, df = 4, *p* = 0.636): However, there is no significant association between IHD onset and gender among male patients (*p* = 0.636), suggesting that the onset of IHD does not vary significantly in this group.

Table 4 provides detailed demographic and clinical data, focusing on main variables such as: age, marital status, social status, type of IHD, and onset of IHD. Below is a breakdown of the main findings:

- Age Distribution
  ○ Female: The largest proportion of female subjects (54.8%) is in the 60–79 age range, followed by 40–59 years (40.5%), and a minority (4.7%) being in the 80–89 age range.
  ○ Male: Most male individuals (63.6%) are younger, in the 40–59 age range; there are fewer individuals in the group of 60–79 years (36.4%), and no males were in the age group of 80–89 years. This suggests that male subjects tend to be younger than female subjects in this cohort.
- Marital Status
  ○ Female: The majority of female patients (53.6%) are married, with 25% being widowed and 21.4% being divorced. There are no single female subjects.
  ○ Male: Similarly, most male patients (69.7%) are married, but unlike females, there are some single males (8.1%). A smaller percentage of males are divorced (19.2%) or widowed (3%). As a result, females have a higher proportion of widows, while males have more singles, suggesting different marital status distributions by gender.
- Social Status
  ○ Female: The largest group of females (44%) is in the age pension category. Other notable categories include disability pension (17.9%), employed (16.7%), handicap pension (11.9%), and unemployed (9.5%).
  ○ Male: In contrast, the majority of male patients (55.6%) fall into the employed category. The distribution across other statuses includes age pension (13.1%), disability pension (13.1%), unemployed (12.1%), and handicap pension (6.1%). Males are more likely to be employed, while females are more represented in the age pension.
- Type of IHD
  ○ Female: The most common types of IHD among female patients are stable and unstable angina pectoris, both at 36.9%. AMI occurs in 23.8% of females, while silent myocardial infarction occurs in 2.4%.
  ○ Male: In contrast, the majority of male patients (52.5%) experienced AMI. Other diagnoses include unstable angina pectoris (23.2%), stable angina pectoris (17.2%), and silent myocardial ischemia (7.1%). This suggests that males are more likely to suffer from AMI compared with females, who are more likely to suffer from angina.
- IHD Onset
  ○ Female: IHD onset in females is relatively evenly distributed across different time intervals. Specifically, 26.2% of females have had IHD for 1–3 months, 20% for over 3 years, 19.1% for 6–12 months and 1–3 years, and 15.5% for 3–6 months. Notably, none of the females have had IHD for less than one month.
  ○ Male: In contrast, males tend to have a shorter disease history. Notably, 41.4% of males have had IHD for 3–6 months, 23.2% for 1–3 months, and 19.2% for 6–12 months. Only 6.1% have had IHD for over 1–3 years or more than 3 years. This indicates that males are more likely to have recent-onset IHD, while females show a more chronic history of the disease lasting over a longer period of time.

**Figure 1:**
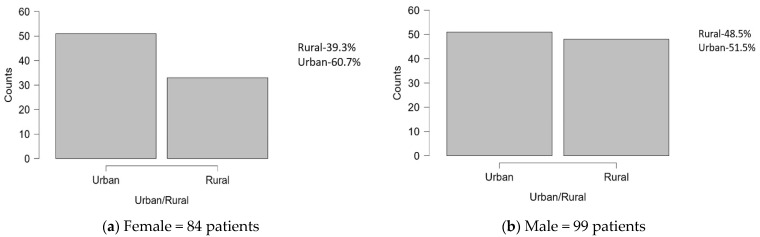
Distribution of subjects by gender and environment of origin.

Figure 1 shows the comparison of gender distribution between urban and rural environments:

- Female: Among the female subjects, the majority (51 subjects, or 60.7%) live in urban areas, while 33 subjects (39.3%) live in rural settings.
- Male: For the male individuals, 51 subjects (51.5%) reside in urban settings, while 48 subjects (48.5%) live in rural areas.

### 3.2. Gender-Based Analysis of Risk Factors

Table 5 presents the distribution of different risk factors among patients, along with corresponding *p*-values indicating the statistical significance of the differences between genders. The following is a breakdown of the main statistics:

- Hypertension
  ○ Female: Among female subjects, 90.5% suffer from hypertension. The *p*-value (<0.001) indicates a significant difference in hypertension prevalence among females.
  ○ Male: In the male group, 96% report having hypertension. The significant *p*-value (<0.001) suggests a strong association between male gender and hypertension prevalence.
- Smoking
  ○ Female: Among the female individuals, 46.4% were smokers. The *p*-value of 0.586 indicates no significant difference in smoking prevalence among female patients.
  ○ Male: In contrast, the majority of male subjects (84.8%) are smokers. The significant *p*-value (<0.001) highlights a strong association between male gender and smoking status.
- Alcohol Abuse
  ○ Female: The majority of female patients (96.4%) reported no alcohol abuse (*p* < 0.001).
  ○ Male: In males, 58.6% report alcohol abuse, although this difference did not reach statistical significance (*p* = 0.107).
- Obesity
  ○ Female: The majority of female patients (76.2%) were classified as obese, with a significant difference (*p* < 0.001).
  ○ Male: In contrast, in males, a percentage of 53.5% were obese, with no significant difference (*p* = 0.547).
- Hypercholesterolemia
  ○ Female: A significant proportion of female subjects (90.5%) had hypercholesterolemia. The *p*-value (<0.001) indicates a significant difference in hypercholesterolemia prevalence.
  ○ Male: Among males, 58.6% reported hypercholesterolemia. The *p*-value of 0.107 suggests that the difference in the prevalence of hypercholesterolemia among men is not statistically significant.
- Hypertriglyceridemia
  ○ Female: Majority of females (85.7%) suffered from hypertriglyceridemia. The *p*-value (<0.001) indicates a significant difference in hypertriglyceridemia rates among females.
  ○ Male: For males, 53.5% have hypertriglyceridemia. The *p*-value of 0.547 shows no significant difference in hypertriglyceridemia rates among males.
- Inflammation
  ○ Female: In female subjects, 72.6% exhibited signs of inflammation. The *p*-value (<0.001) indicates a significant prevalence of inflammation among females.
  ○ Male: Among males, a significantly high proportion (72.7%) show signs of inflammation. The *p*-value (<0.001) suggests that inflammation is a critical risk factor.
- Tachycardia
  ○ Female: Tachycardia is present in 60.7% of females. The *p*-value of 0.063 indicates that the difference was not statistically significant.
  ○ Male: In males, tachycardia occurs in 54.5%. The *p*-value (0.422) suggests no significant difference in tachycardia prevalence among males.
- Genetic Factors
  ○ Female: Genetic factors are present in 70.2% of females. The *p*-value (<0.001) indicates a significant association with gender.
  ○ Male: Among males, 57.6% reported genetic factors. The *p*-value of 0.159 suggests no significant difference in genetic factors among males.

### 3.3. Gender-Based Differences in Emotional, Social, and Behavioral Responses to IHD

Table 6 provides a descriptive analysis of the psychological and behavioral impact of IHD, detailing their responses to various questions regarding their emotional, social, and physical health. The key statistics are interpreted as follows:

- Emotional response to diagnosis (Q1)
  ○ Female: The majority of females (66.6%) report feeling deep sadness that they cannot seem to overcome, while 27.4% feel overwhelmed by sadness. Only 1.2% feel good about managing their condition.
  ○ Male: Similarly, most male subjects (44.5%) feel overwhelmed by sadness, but a slightly lower proportion (42.4%) report deep sadness compared with females. A minority (3%) feel comfortable about managing their condition. Both genders express significant emotional distress following diagnosis, but females report a slightly higher prevalence of deep sadness compared with males.
- Changes in frustration or anger (Q2)
  ○ Female: More than half of female subjects (57.1%) notice that they become frustrated more easily, while 42.9% feel anger quickly rises in response to small triggers. No females report feeling angry all the time.
  ○ Male: Males show similar levels of frustration (49.5%) and anger (39.4%). However, 8.1% of males feel angry all the time about their health problems. Both genders experience increased frustration and anger, but males are more likely to report persistent anger.
- Communication ability (Q3)
  ○ Female: Many females (39.3%) report feeling indifferent to social interactions, while 28.6% prefer solitude. Only 3.5% feel their communication abilities remain unchanged.
  ○ Male: Almost half of male patients (49.5%) find it more difficult to express their emotions, and a similar proportion (26.2%) prefer solitude. More males (6.1%) report unchanged communication ability compared with females. Both genders experience challenges in communication post-diagnosis, with a higher percentage of males reporting difficulty expressing emotions.
- Work performance and motivation (Q4)
  ○ Female: Most females (64.3%) feel unable to perform work responsibilities, with only 14.3% exerting extra effort. Only 1.2% maintain consistent work performance.
  ○ Male: Similarly, 60.6% of males report being unable to perform work responsibilities, with 19.2% needing extra effort. Both genders face significant declines in work performance and motivation post-diagnosis, with slightly more males needing extra effort to maintain work responsibilities.
- Future outlook (Q5)
  ○ Female: The majority of females (57.1%) feel hopeless about their future health, with only 3.6% maintaining optimism.
  ○ Male: Male subjects also report a high level of hopelessness (38.4%), but more males (5%) express optimism about their future compared with females. Females exhibit higher levels of hopelessness about their future health compared with males, although both genders display significant pessimism.
- Self-harm and suicidal thoughts (Q6)
  ○ Female: While 33.3% of females have never thought about self-harm, 44% occasionally think about it, and 17.9% would consider ending their life.
  ○ Male: A slightly higher proportion of males (44.4%) have never considered self-harm, while 39.4% occasionally think about it. Fewer males (10.1%) would consider ending their life. Females report a higher prevalence of suicidal thoughts compared with males.
- Sleep quality (Q7)
  ○ Female: More than half of females (57.1%) often wake up early and cannot return to sleep, and only 2.4% report sleeping as well as before their diagnosis.
  ○ Male: Similarly, 34.4% of males report waking up early, while 32.3% have trouble sleeping. Only 3% report no change in sleep quality. Both genders experience significant sleep disturbances, with females reporting more frequent difficulty in returning to sleep.
- Energy levels and fatigue (Q8)
  ○ Female: Most females (67.8%) feel so exhausted that they struggle to do anything, while only 2.4% feel as energetic as before.
  ○ Male: A lower proportion of males (46.5%) report extreme exhaustion, with 38.4% getting tired easily. Females report more fatigue compared with males, though both genders experience a decline in energy levels.
- Appetite changes (Q9)
  ○ Female: More than half (52.3%) of females report having little or no appetite, with only 3.6% maintaining the same appetite as before.
  ○ Male: A smaller percentage (28.3%) of males report a lack of appetite, and 9.1% experience no change in appetite. Females experience more pronounced appetite loss compared with males post-diagnosis.
- Health concerns (Q10)
  ○ Female: A large proportion of females (70.2%) are consumed by worry about their health, while 7.1% report no concerns.
  ○ Male: Fewer males (46.5%) are consumed by health worries, and 5% report no concerns. Females show higher levels of anxiety and concern regarding their health compared with males.
- Interest in romantic relationships (Q11)
  ○ Female: The majority of females (64.3%) have completely lost interest in romantic connections, with only 3.6% maintaining the same interest as before.
  ○ Male: Fewer males (30.3%) report a complete loss of interest, while 11.1% feel their romantic interests remain unchanged. Females report a greater loss of interest in romantic relationships compared with males.

Table 7 presents the chi-square test results for gender differences in emotional, social, and behavioral responses to IHD. It includes chi-square values, degrees of freedom, and *p*-values for each question, indicating whether gender influences various aspects of living with IHD. Significant findings suggest gender-based differences, while non-significant results indicate similar impacts across genders.

- Significant findings (*p* < 0.05)
  ○ Q1 (emotional reactions to IHD diagnosis): significant gender differences in emotional responses (*p* = 0.012), with males and females expressing sadness differently.
  ○ Q2 (frustration or anger): gender differences in frustration or anger (*p* = 0.019), suggesting varying emotional responses.
  ○ Q3 (changes in communication): significant differences in communication patterns (*p* = 0.005), with varying difficulties in social interactions by gender.
  ○ Q7 (sleep quality): significant gender differences in sleep disturbances (*p* = 0.015), with one gender experiencing more severe disruptions.
  ○ Q8 (fatigue and energy levels): gender differences in fatigue and energy (*p* = 0.005), indicating distinct physical impacts of IHD.
  ○ Q9 (changes in appetite): significant gender differences in appetite changes (*p* = 0.003).
  ○ Q10 (concerns about health and its impact): significant differences in concerns about health and its effects on life (*p* = 0.002).
  ○ Q11 (interest in romantic relationships or intimacy): highly significant gender differences in interest in relationships (*p* < 0.001).
- Non-significant findings (*p* ≥ 0.05)
  ○ Q4 (work performance and motivation): no significant gender differences in work performance (*p* = 0.853), indicating similar effects on professional life.
  ○ Q5 (perspectives on the future): no significant gender differences in future perspectives (*p* = 0.084).
  ○ Q6 (consideration of self-harm or suicide): no significant gender differences in considerations of self-harm (*p* = 0.283).

### 3.4. Scale and Item-Level Reliability Analysis

The scale’s internal consistency was evaluated using Cronbach’s alpha (α). The overall point estimate for Cronbach’s α was 0.915, with a 95% confidence interval ranging from 0.896 to 0.931 (Table 8), suggesting a high level of internal consistency. Pairwise complete cases were used in the analysis to handle missing data.

The reliability of each individual item was also assessed using the “if item dropped” statistic. Cronbach’s α-values for the scale if each item was removed ranged from 0.897 to 0.916 (Table 9). None of the items, when removed, led to a substantial change in the overall α-value. The lowest α observed when an item was dropped was 0.897 for Q10, and the highest was 0.916 for Q2. This suggests that removing any of the individual items does not markedly affect the reliability of the scale.

### 3.5. Assessing the Willingness for Psychological or Psychiatric Support Among IHD Patients

**Figure 2:**
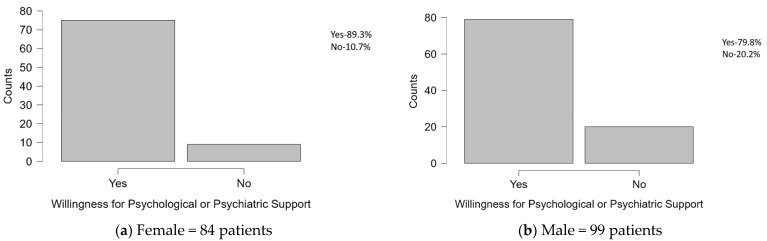
Willingness of patients to seek emotional support for IHD.

Figure 2 illustrates the willingness of patients to seek emotional support within the study population, measured by the last question in the questionnaire. Below is the interpretation of the key statistics:

- Female: Of 84 female subjects, 75 (89.3%) expressed willingness to receive emotional support. This high level of willingness indicates that the majority of female patients are open to seeking psychological/psychiatric support services to cope with IHD.
- Male: In contrast, 79 (79.8%) out of 99 male patients expressed willingness to receive emotional support. This finding reveals that a notable majority of male patients also are open to seek psychological/psychiatric support, although it is a lower percentage compared with their female counterparts.

### 3.6. Distribution of Depression Grades in the Patient Population

**Figure 3:**
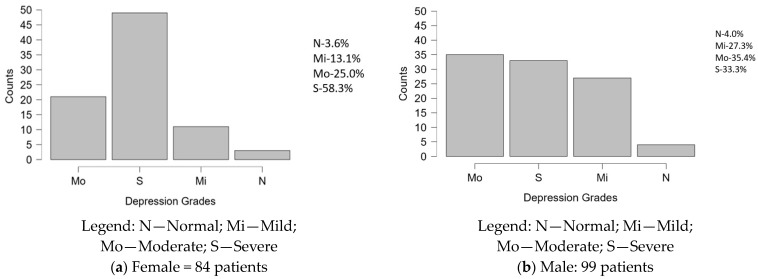
Subject’s grade of depression.

Figure 3 illustrates the distribution of depression grades within the study population based on the scores calculated from the self-developed questionnaire. The interpretation of the key statistics is outlined below:

- Female: Among the female, a small number of subjects (3 or 3.6%) are in remission (N), while 11 (13.1%) individuals experience mild depression (Mi). A larger group of 21 (25.0%) individuals are classified as moderately depressed (Mo), but the majority (49, or 58.3% subjects) of individuals are experiencing severe depression (S).
- Male: Among males, 4 (4%) subjects are in remission (N) and 27 (27.3%) have mild depression (Mi). A larger number of 35 (35.4%) individuals are classified as moderately depressed (Mo), while 33 (33.3%) subjects experience severe depression (S). Compared with females, a greater proportion of males exhibit mild to moderate depression, though the prevalence of severe depression remains high in both genders.

## 4. Discussion

The findings of this pilot study provide important insights into the demographic, clinical, psychological, and psychiatric profile of male and female patients admitted to the Psychiatry Department at Arad County Emergency Hospital, Romania, and diagnosed with depression and IHD. Gender differences in both the emotional and social responses to IHD, as well as the wider implications for treatment and care, highlight the complexity of managing these patients.

### 4.1. Demographic and Clinical Characteristics

Age and social status significantly differed between male and female patients. Females in this cohort tended to be older, with 59.5% being over the age of 60, while males were younger, with the majority (63.6%) being in the 40–59 age range. These differences align with the broader literature, suggesting that IHD tends to occur later in life for females due to the cardioprotective effects of estrogen before menopause [29,30]. Social status also varied by gender, with a higher proportion of females being classified in the age pension status and more males being classified as employed. This may reflect socioeconomic challenges faced by women, particularly older women, potentially impacting their access to healthcare and their ability to manage chronic conditions [31,32].

In terms of IHD type, males were more frequently diagnosed with AMI (52.5%), while females presented with stable angina (36.9%) or unstable angina (36.9%). These results suggest that females may experience a more chronic progression of IHD, whereas males are more likely to experience acute, life-threatening events [8]. Interestingly, IHD onset differed significantly between the genders, with males more often being diagnosed within a shorter time frame (41.4% within 3–6 months), while females had a more prolonged disease onset. This may suggest potential delays in diagnosis and treatment for women, which could be an area for further investigation [33,34].

### 4.2. Psychological and Behavioral Impact

The emotional response to an IHD diagnosis differed markedly between males and females, with women reporting a higher level of sadness and hopelessness. Notably, 66.6% of females expressed deep, unrelenting sadness, compared with 42.4% of males. This is consistent with previous research showing that women with cardiovascular disease are more likely to experience depression [14,35]. Depression severity was also higher in females, with the majority being classified as severely depression, while males exhibited a more even distribution between mild and moderate depression. This highlights the importance of integrating psychological and psychiatric interventions, particularly for female patients who may struggle more with the emotional impact of IHD [36,37].

Fatigue, a common symptom often linked to depression, was reported by 67.8% of females and 46.5% of males in this study. The strong association between fatigue and depression in this cohort warrants further attention. Fatigue not only serves as a physical manifestation of emotional distress but also amplifies the symptoms of depression. In many IHD patients, especially women, fatigue creates a vicious cycle where physical exhaustion worsens depressive feelings and vice versa [38]. This interplay emphasizes the importance of a comprehensive treatment approach that addresses both the psychological and physical components of fatigue. Integrating targeted management strategies, including both pharmacological and non-pharmacological interventions, is crucial in breaking this cycle and improving overall patient outcomes [39,40].

Similarly, frustration and anger were common across both genders, although anger levels were higher in males, with 8.1% reporting constant anger compared with none among females. This difference may be related to traditional gender roles and coping mechanisms, where men externalize their emotions as anger, whereas women internalize their emotional distress as sadness [41,42].

### 4.3. Social Functioning and Communication

The impact of IHD on social functioning and communication was also significant, with females being more likely to withdraw from social interactions, express indifference towards communication (39.3%), or prefer solitude (28.6%). In contrast, 49.5% of males found it more difficult to express feelings post-diagnosis but did not retreat from social contact as significantly as women. These patterns suggest that females may experience more social isolation as a result of their illness, which could exacerbate their mental health challenges [43,44]. The findings highlight the need for a gender-sensitive approach in managing the social and emotional well-being of IHD patients [45,46].

### 4.4. Work Performance and Motivation

Both males and females reported a significant decline in their work performance. More than 60% of both groups reported feeling unable to perform their job responsibilities after their diagnosis. This decline in occupational performance, combined with high levels of fatigue (67.8% in females, 46.5% in males), indicates that IHD significantly impacts daily life and productivity. These data suggest that rehabilitation programs should include vocational support with an emphasis on gradual return to or adjustment to work to reduce the psychological and physical burden on IHD patients [47,48].

### 4.5. Health Concerns, Appetite, and Sleep

This study also revealed significant concerns regarding future health, with 57.1% of females and 38.4% of males feeling hopeless about their prognosis. This feeling of hopelessness was accompanied by changes in appetite and sleep patterns. The majority of females reported significant loss of appetite (52.3%), and sleep disturbances were more prevalent in women, with 57.1% often waking early and being unable to return to sleep. Sleep and appetite disorders, common in depression, may further complicate the management of IHD, particularly in females. Changes in lifestyle, coupled with time constraints and irregular eating patterns, contribute to the increased consumption of processed foods and sweetened beverages, which can lead to obesity and is associated with higher rates of depression [49,50,51]. These findings highlight the need for comprehensive care that addresses not only the physical but also the emotional and psychosocial aspects of the disease [52,53].

### 4.6. Inflammation Effects

The findings of this study underscore the significant role of inflammation in the context of ischemic heart disease (IHD). A high prevalence of inflammatory markers was observed in both male (72.7%) and female (72.6%) patients, highlighting the importance of chronic inflammation in the progression of IHD. This is particularly concerning because chronic, long-term inflammation is known to contribute to the worsening of cardiovascular conditions and is associated with poorer outcomes in IHD patients [54].

However, it is important to recognize that inflammation has different implications depending on the timing and stage of the disease. For example, while chronic inflammation can exacerbate IHD and contribute to its progression, the inflammatory response that occurs immediately after an AMI serves a different function. Inflammation in the acute phase is part of the body’s natural healing process, aiding tissue repair and recovery [55,56]. This response typically lasts for several days to weeks, but if it becomes prolonged or dysregulated, it can lead to further cardiovascular damage and worsen outcomes. Prolonged acute inflammation may also affect other systems in the body, such as the brain, contributing to neuroinflammation and potentially influencing mental health [57].

The link between chronic inflammation and depression is particularly relevant in IHD patients. Inflammatory cytokines, such as IL-6 and TNF-alpha, are believed to influence brain chemistry and mood regulation, playing a key role in the development of depression [58,59]. This bidirectional relationship—where chronic inflammation exacerbates IHD and depression further worsens cardiovascular health—complicates the management of both physical and mental health in these patients [60,61].

Moreover, the resistance to current antimicrobial therapies observed in IHD patients, often accompanied by increased rates of depression and anxiety, further emphasizes the need for comprehensive management strategies. Chronic inflammation not only exacerbates cardiovascular damage but also impairs immune responses, leading to microbial resistance. This resistance, coupled with higher rates of depression and anxiety, highlights the need for more effective treatment approaches that address both the physical and mental health aspects of IHD. Addressing inflammation in both its acute and chronic forms could have a positive impact on physical and mental health outcomes. This suggests a potential avenue for improving treatment efficacy in IHD patients. Therefore, there is an urgent need for further research into targeted anti-inflammatory therapies and integrated care approaches that address both the cardiovascular and psychiatric aspects of IHD [62,63].

In summary, while inflammation serves a protective role immediately after an acute event like AMI, chronic inflammation remains a significant risk factor for both IHD progression and depression. Future research should focus on understanding the complex relationship between inflammation, IHD, and depression to improve clinical strategies for managing these intertwined conditions [64,65].

### 4.7. Gender-Specific Interventions and Future Directions

One of the most notable findings from this study is the willingness of both genders to seek emotional support, although women were slightly more open to emotional support than men (88.1% vs. 79.8%). This openness represents a valuable opportunity for healthcare providers to integrate mental health services into the standard cardiac care, particularly targeting women who experience greater emotional distress. Customized psychotherapeutic interventions, such as cognitive behavioral therapy and support groups, may significantly improve treatment outcomes [66,67].

Future research should focus on the early detection and treatment of psychiatric symptoms in IHD patients, especially considering the higher rates of depression and hopelessness in female patients. Additionally, healthcare systems should consider adopting a multidisciplinary approach that combines cardiology, psychiatry, and social work to address the comprehensive needs of these patients.

Furthermore, future studies should investigate the potential role of anterior chest wall conformation in influencing IHD prevalence and outcomes. Research suggests that individuals with concave chest wall structures often have distinct clinical and psychological profiles, including a predisposition to anxiety disorders, which may impact cardiovascular health. Investigating the interplay between anatomical features, psychological factors, and IHD progression could offer critical insights into personalized care strategies, particularly in the context of gender-specific risks. Such research could ultimately improve clinical decision making and patient outcomes [22].

### 4.8. Practical Applications in the Field

The findings of this study have several practical implications for improving the management and care of patients with IHD and comorbid depression, particularly when considering gender differences.

1. Tailored mental health interventions: The higher prevalence of severe depression and hopelessness among female patients underscores the need for gender-sensitive mental health interventions in the context of IHD. Healthcare providers should prioritize integrating psychiatric care, such as counseling or cognitive behavioral therapy, into routine cardiac care, especially for female patients who face greater emotional challenges.
2. Comprehensive cardiac rehabilitation programs: This study highlights the importance of addressing psychological symptoms such as fatigue, frustration, and social withdrawal in cardiac rehabilitation. Programs should include elements such as stress management, support groups, and vocational rehabilitation to support the emotional, social, and occupational recovery of IHD patients.
3. Gender-specific health policies: Policy makers and healthcare systems can leverage these findings to develop gender-specific health policies aimed at improving access to early diagnosis and treatment, particularly for women who may experience delayed care or chronic disease progression. This includes improving education about IHD symptoms that are more common in women, such as fatigue and stable angina.
4. Integration of inflammation management: The role of chronic inflammation in exacerbating both depression and IHD suggests that anti-inflammatory therapies should be further explored and integrated into treatment plans. Addressing inflammation through dietary interventions, physical activity, or pharmacological approaches could enhance both physical and mental health outcomes.
5. Training for healthcare providers: The insights from this study can be used to inform the training of healthcare professionals on the interplay between cardiovascular and psychological health, emphasizing the importance of a multidisciplinary approach. Cardiologists, psychiatrists, and social workers should work collaboratively to address the complex needs of these patients.

### 4.9. Research Limitations

Despite the important insights provided by this study, several limitations need to be recognized. The descriptive, cross-sectional design only provides a snapshot of patients’ psychological and social responses to IHD at a single point in time, limiting the ability to draw causal inferences. Longitudinal studies are needed to trace how these factors evolve and influence long-term outcomes.

Second, the study did not consider the influence of anterior chest wall conformation on IHD prevalence and prognosis. This may be a significant limitation, as evidence indicates that individuals with concave chest wall anatomies exhibit lower IHD prevalence and distinct clinical outcomes. These individuals are often associated with anxiety disorders and other psychological challenges, which could further influence cardiovascular health [22]. Addressing this gap in future research could provide a more comprehensive understanding of the interplay between anatomical and psychological factors in IHD, ultimately helping to refine diagnostic and treatment approaches.

Third, the sample size was relatively small, and the study was conducted at a single hospital in Romania, which may limit the generalizability of the findings. Expanding the research to multiple hospitals and more diverse populations would enhance its broader relevance. Additionally, while the self-developed questionnaire demonstrated strong internal consistency (Cronbach’s α = 0.915), its lack of external validation raises concerns about its reliability. Future studies should aim to validate this tool in larger and more diverse populations.

Lastly, important factors such as medication adherence, healthcare access, and cultural attitudes toward the disease were not investigated, though they may contribute to the gender differences observed. These variables should be explored in future research to offer a more comprehensive understanding of IHD outcomes.

## 5. Conclusions

This pilot study provides valuable insights into gender differences in the psychological and social responses to IHD among patients admitted to a psychiatry department. Women in this cohort exhibited more profound emotional distress, social isolation, and significant appetite and sleep disturbances, while men demonstrated higher levels of anger and frustration. These findings underscore the complex, multifaceted impact of IHD on mental health, with each gender manifesting different emotional and behavioral challenges.

This study highlights the critical need for gender-specific interventions beyond traditional cardiac care to address these psychological, psychiatric, social, and behavioral differences. For women, a focus on managing depression, addressing social isolation, and improving sleep and appetite may lead to better overall outcomes. For men, interventions targeting anger management, emotional expression, and coping strategies may be necessary to reduce frustration. In both cases, personalized care plans that integrate mental health services into cardiac rehabilitation may be important for improving recovery and quality of life.

Furthermore, this study emphasizes the importance of a multidisciplinary approach integrating cardiology, psychiatry, psychology, and social work to ensure a holistic treatment plan. Mental healthcare should be a central component of cardiac rehabilitation, and regular screening for depression and anxiety should be performed, especially for high-risk groups such as postmenopausal women and young men with acute forms of IHD.

Future research should prioritize longitudinal studies to explore the long-term impacts of gender-specific interventions and the role of mental health in the prognosis of IHD patients. Expanding the sample size and geographic scope will also help confirm these findings and support the development of more generalizable, evidence-based interventions that address both the physical and emotional health of IHD patients. In this way, healthcare providers could adopt a more comprehensive, gender-sensitive approach to managing IHD and its associated psychological burdens, ultimately improving patient outcomes and quality of life.

## Figures and Tables

**Table 1:** Inclusion and exclusion criteria.

Inclusion Criteria	Exclusion Criteria
Adult patients (18 years or older)	Patients with a pre-existing diagnosis of severe psychiatric disorders
Patients of both genders	Patients with severe cognitive impairment
Patients admitted to Arad County Emergency Clinical Hospital, Romania, Psychiatry Department	Patients with severe comorbid medical conditions (other than IHD)
Patients confirmed with IHD, depression	Patients with no evidence of diagnosis of IHD according to diagnostic guidelines
Patients who provided informed consent to participate in the study	Patients who do not provide informed consent to participate

**Table 2:** Grade of depression evaluated after applying the questionnaire.

Total Score	Depression Grade
0–8	Normal
9–16	Mild
17–24	Moderate
25–33	Severe

**Table 3:** Multinomial test results for gender differences in demographic and clinical variables.

	Female = 84 Patients			Male = 99 Patients		
Variable	χ^2^	Degrees of Freedom	*p*	χ^2^	Degrees of Freedom	*p*
Age	33.429	2	<0.001	7.364	1	<0.001
Social status	32.310	4	<0.001	79.939	4	<0.001
Marital status	15.643	2	<0.001	110.899	3	<0.001
Type of ischemic heart disease	26.762	3	<0.001	45.283	3	<0.001
Ischemic heart disease onset	62.152	5	<0.001	2.548	4	0.636

Statistical method: multinomial test.

**Table 4:** Descriptive analysis of demographic and clinical variables across gender.

		Female = 84 Patients	Male = 99 Patients
Variable	Level	Counts	Counts
Age	40–59	34 (40.5%)	63 (63.6%)
	60–79	46 (54.8%)	36 (36.4%)
	80–89	4 (4.7%)	0 (0%)
Marital Status	Divorced	18 (21.4%)	19 (19.2%)
	Married	45 (53.6%)	69 (69.7%)
	Single	0 (0%)	8 (8.1%)
	Widowed	21 (25%)	3 (3%)
Social Status	Age pension	37 (44%)	13 (13.1%)
	Disability pension	15 (17.9%)	13 (13.1%)
	Employed	14 (16.7%)	55 (55.6%)
	Extra disability benefits	10 (11.9%)	6 (6.1%)
	Unemployed	8 (9.5%)	12 (12.1%)
Type of ischemic heart disease		20 (23.8%)	52 (52.5%)
	Stable angina pectoris	31 (36.9%)	17 (17.2%)
	Silent myocardial infarction	2 (2.4%)	7 (7.1%)
	Unstable angina pectoris	31 (36.9%)	23 (23.2%)
Ischemic heart disease onset	<1 month	0 (0%)	4 (4%)
	1–3 months	22 (26.2%)	23 (23.2%)
	3–6 months	13 (15.5%)	41 (41.4%)
	6–12 months	16 (19.1%)	19 (19.2%)
	1–3 years	16 (19.1%)	6 (6.1%)
	>3 years	17 (20%)	6 (6.1%)

Statistical method: descriptive analysis.

**Table 5:** Binomial test application for risk factors across gender.

		Female = 84 Patients		Male = 99 Patients	
Variable	Level	Counts	*p*	Counts	*p*
Hypertension	No	8 (9.5%)	<0.001	4 (4%)	<0.001
	Yes	76 (90.5%)	<0.001	95 (96%)	<0.001
Smoking	No	45 (53.6%)	0.586	15 (15.2%)	<0.001
	Yes	39 (46.4%)	0.586	84 (84.8%)	<0.001
Alcohol abuse	No	81 (96.4%)	<0.001	41 (41.4%)	0.107
	Yes	3 (3.6%)	<0.001	58 (58.6%)	0.107
Obesity	No	20 (23.8%)	<0.001	46 (46.5%)	0.547
	Yes	64 (76.2%)	<0.001	53 (53.5%)	0.547
Hypercholesterolemia	No	8 (9.5%)	<0.001	41 (41.4%)	0.107
	Yes	76 (90.5%)	<0.001	58 (58.6%)	0.107
Hypertriglyceridemia	No	12 (14.3%)	<0.001	46 (46.5%)	0.547
	Yes	72 (85.7%)	<0.001	53 (53.5%)	0.547
Inflammation	No	23 (27.4%)	<0.001	27 (27.3%)	<0.001
	Yes	61 (72.6%)	<0.001	72 (72.7%)	<0.001
Tachycardia	No	33 (39.3%)	0.063	45 (45.5%)	0.422
	Yes	51 (60.7%)	0.063	54 (54.5%)	0.422
Genetic factors	No	25 (29.8%)	<0.001	42 (42.4%)	0.159
	Yes	59 (70.2%)	<0.001	57 (57.6%)	0.159

Statistical method: binomial test.

**Table 6:** Descriptive analysis of emotional, social, and behavioral responses to IHD by gender.

		Female 84 Patients	Male 99 Patients
Variable	Level	Counts	Counts
Q1. How did you feel after being diagnosed with IHD?	0. I feel good about managing my condition	1 (1.2%)	3 (3%)
	1. Sometimes I feel sad about my diagnosis	4 (4.8%)	10 (10.1%)
	2. I often feel overwhelmed by sadness due to my heart condition	23 (274%)	44 (44.5%)
	3. I feel a deep sadness that I can’t seem to overcome	56 (66.6%)	42 (42.4%)
Q2. Since your diagnosis, how has your frustration or anger changed?	0. I am managing my emotions as I did before	0 (0%)	3 (3%)
	1. I have noticed that I get frustrated more easily than before	48 (57.1%)	49 (49.5%)
	2. I feel anger rising quickly in response to small triggers	36 (42.9%)	39 (39.4%)
	3. I feel angry all the time about my health problems	0 (0%)	8 (8.1%)
Q3. How has your ability to communicate with others changed since your diagnosis?	0. I communicate as well as I did before my diagnosis	3 (3.5%)	6 (6.1%)
	1. I find it more difficult to express my feelings to others	24 (28.6%)	49 (49.5%)
	2. I feel indifferent to social interactions now	33 (39.3%)	18 (18.2%)
	3. I prefer solitude and avoid communication with others	24 (28.6%)	26 (26.2%)
Q4. Have you noticed any changes in your work performance or motivation?	0. My work performance is consistent with pre-diagnosis levels	1 (1.2%)	1 (1%)
	1. I need to exert extra effort to perform my job	12 (14.3%)	19 (19.2%)
	2. I struggle to motivate myself to work	17 (20.2%)	19 (19.2%)
	3. I feel unable to perform my work responsibilities	54 (64.3%)	60 (60.6%)
Q5. How do you envision your future in light of your heart condition?	0. I am optimistic about my future health and well-being	3 (3.6%)	5 (5%)
	1. It’s challenging for me to think positively about the future	13 (15.5%)	25 (25.3%)
	2. I have low expectations regarding my health and future	20 (23.8%)	31 (31.3%)
	3. I feel hopeless about what lies ahead due to my illness	48 (57.1%)	38 (38.4%)
Q6. Since your diagnosis, have you ever considered self-harm or suicide?	0. I have never thought about harming myself	28 (33.3%)	44 (44.4%)
	1. I have occasionally thought about it	37 (44%)	39 (39.4%)
	2. I find myself wishing to harm myself	4 (4,8%)	6 (6.1%)
	3. If given the opportunity, I would consider ending my life	15 (17.9%)	10 (10.1%)
Q7. How would you describe your sleep quality since being diagnosed with IHD?	0. I sleep as well as before my diagnosis	2 (2.4%)	3 (3%)
	1. I have trouble sleeping as I used to	14 (16.7%)	32 (32.3%)
	2. I frequently wake up early and struggle to fall back asleep	20 (23.8%)	30 (30.3%)
	3. I often wake up early and cannot return to sleep	48 (57.1%)	34 (34.4%)
Q8. Have you noticed changes in your energy levels or fatigue?	0. I feel as energetic as I did before	2 (2.4%)	0 (0%)
	1. I notice I get tired more quickly than before	10 (11.9%)	15 (15.1%)
	2. I feel tired even with minimal activity	15 (17.9%)	38 (38.4%)
	3. I feel so exhausted that I struggle to do anything	57 (67.8%)	46 (46.5%)
Q9. Have you experienced changes in your appetite since your diagnosis?	0. My appetite is the same as before	3 (3.6%)	9 (9.1%)
	1. My appetite has decreased somewhat	13 (15.5%)	31 (31.3%)
	2. I eat less than I did before	24 (28.6%)	31 (31.3%)
	3. I have little to no appetite	44 (52.3%)	28 (28.3%)
Q10. How concerned are you about your overall health and its impact on your life?	0. I feel fine and have no worries	6 (7.1%)	5 (5%)
	1. I have some concerns about my condition	3 (3.6%)	16 (16.2%)
	2. My health problems overwhelm my thoughts and feelings	16 (19.1%)	32 (32.3%)
	3. I am too consumed by worry to focus on anything else	59 (70.2%)	46 (46.5%)
Q11. Have you noticed changes in your interest in romantic relationships or intimacy?	0. My feelings toward romantic interests remain unchanged	3 (3,6%)	11 (11.1%)
	1. I feel less interested in romantic relationships than before	10 (11.9%)	27 (27.3%)
	2. I have become significantly less interested in intimacy	17 (20.2%)	31 (31.3%)
	3. I have completely lost interest in romantic connections	54 (64.3%)	30 (30.3%)

Statistical method: descriptive analysis

**Table 7:** Chi-square test results for gender differences in emotional, social, and behavioral responses to IHD diagnosis.

Variable	Value	Degrees of Freedom	*p*
Q1. How did you feel after being diagnosed with IHD?	10.998	3	0.012
Q2. Since your diagnosis, how has your frustration or anger changed?	9.968	3	0.019
Q3. How has your ability to communicate with others changed since your diagnosis?	12.911	3	0.005
Q4. Have you noticed any changes in your work performance or motivation?	0.783	3	0.853
Q5. How do you envision your future in light of your heart condition?	6.640	3	0.084
Q6. Since your diagnosis, have you ever considered self-harm or suicide?	3.804	3	0.283
Q7. How would you describe your sleep quality since being diagnosed with IHD?	10.475	3	0.015
Q8. Have you noticed changes in your energy levels or fatigue?	13.014	3	0.005
Q9. Have you experienced changes in your appetite since your diagnosis?	13.672	3	0.003
Q10. How concerned are you about your overall health and its impact on your life?	14.798	3	0.002
Q11. Have you noticed changes in your interest in romantic relationships or intimacy?	22.243	3	<0.001
Total	183		

Statistical method: chi-square test.

**Table 8:** Scale reliability statistics.

Estimate	Value
Point estimate	0.915
95% confidence interval lower bound	0.896
95% confidence interval upper bound	0.931

Statistical method: Cronbach’s α Test.

**Table 9:** Individual item reliability statistics.

Item	Value
Q1. How did you feel after being diagnosed with IHD?	0.913
Q2. Since your diagnosis, how has your frustration or anger changed?	0.916
Q3. How has your ability to communicate with others changed since your diagnosis?	0.911
Q4. Have you noticed any changes in your work performance or motivation?	0.905
Q5. How do you envision your future in light of your heart condition?	0.898
Q6. Since your diagnosis, have you ever considered self-harm or suicide?	0.910
Q7. How would you describe your sleep quality since being diagnosed with IHD?	0.903
Q8. Have you noticed changes in your energy levels or fatigue?	0.907
Q9. Have you experienced changes in your appetite since your diagnosis?	0.914
Q10. How concerned are you about your overall health and its impact on your life?	0.897
Q11. Have you noticed changes in your interest in romantic relationships or intimacy?	0.901

Statistical method: Cronbach’s α if item dropped.

## Data Availability

The raw data supporting the conclusions of this article will be made available by the authors on request.
